# Research of Fall Detection and Fall Prevention Technologies: A Review

**DOI:** 10.3390/s26041192

**Published:** 2026-02-12

**Authors:** Dan Hrubý, Eva Hrubá, Martin Černý

**Affiliations:** Faculty of Electrical Engineering and Computer Science, VSB—Technical University of Ostrava, 17. listopadu 15, 708 33 Ostrava-Poruba, Czech Republic; eva.hruba@vsb.cz (E.H.); martin.cerny@vsb.cz (M.Č.)

**Keywords:** fall detection, wearable fall detectors, unobtrusive fall detectors

## Abstract

Falls represent a significant global public health issue, particularly among adults over the age of 60. This comprehensive review aims to provide an in-depth examination of current fall detection and prevention technologies. The study categorizes fall detection methods into pre-fall prediction and post-fall detection, using both wearable and unobtrusive sensors. Wearable technologies, such as accelerometers, gyroscopes, and electromyography (EMG) sensors, are explored for their efficacy in real-time fall prediction and detection. Unobtrusive methods, including camera-based systems, LiDAR, radar, ultrasonic sensors, and depth sensors, are evaluated for their ability to monitor falls without intruding on users’ daily activities. The integration of these technologies into healthcare settings is also discussed, with an emphasis on the importance of immediate response to fall events. By analyzing the operational principles, technological advancements, and practical applications of these systems, promising directions for future research and innovation in fall detection and prevention are identified. The findings highlight the need for multifaceted approaches combining various sensor technologies to enhance fall detection accuracy and response times, ultimately improving patient safety and quality of life.

## 1. Introduction

Falls represent an important global public health issue and are considered the second leading cause of fatal unintentional injuries worldwide. Every year, it is estimated that 684,000 people lose their lives due to falls, with more than 80% of these deaths occurring in low- and middle-income countries [1,2]. Among the affected population, adults over the age of 60 suffer the highest number of fatal falls. Furthermore, falls among older people are a pressing issue, and the statistics are alarming. The World Health Organization reports that about 28–35% of people aged 65 and over experience a fall every year, with this percentage rising to 32–42% for people over 70 [1]. In addition, without immediate preventive measures, the number of injuries resulting from falls is expected to double by 2030. These falls not only cause physical injuries but also trigger a cycle of fear of falling, leading to reduced physical activity, social isolation and a decreased quality of life [1,2,3].

### Brief Pathophysiology of Fall

With aging, declines in muscle strength, proprioception, vision, and vestibular function impair the ability to generate corrective torques, increasing susceptibility to center-of-mass displacement beyond the base of support [4]. Cognitive impairments—most notably diminished processing speed and executive function—adversely affect the timely integration of multisensory information and motor planning, resulting in an increased risk of balance instability [5]. Gait abnormalities such as decreased speed, increased stride-time variability, and reduced swing-phase control also signal an unstable locomotor pattern that predisposes to trips and slips [6]. Chronic medical conditions (e.g., Parkinson’s disease, peripheral neuropathy, osteoarthritis) and polypharmacy (especially sedatives and antihypertensives) exacerbate these physiological deficits by altering neuromuscular coordination and causing orthostatic hypotension, which together heighten fall risk [5]. Environmental factors—poor lighting, cluttered walkways, and uneven flooring—act as external triggers that interact with the individual’s compromised balance system, converting a near-miss into a completed fall [4,5]. These multifactorial mechanisms underline the need for fall detection and prevention technologies that capture not only gross body motion, but also subtle changes in gait, posture, and response latency, motivating the development of both wearable and unobtrusive sensing approaches.

**Figure 1:**
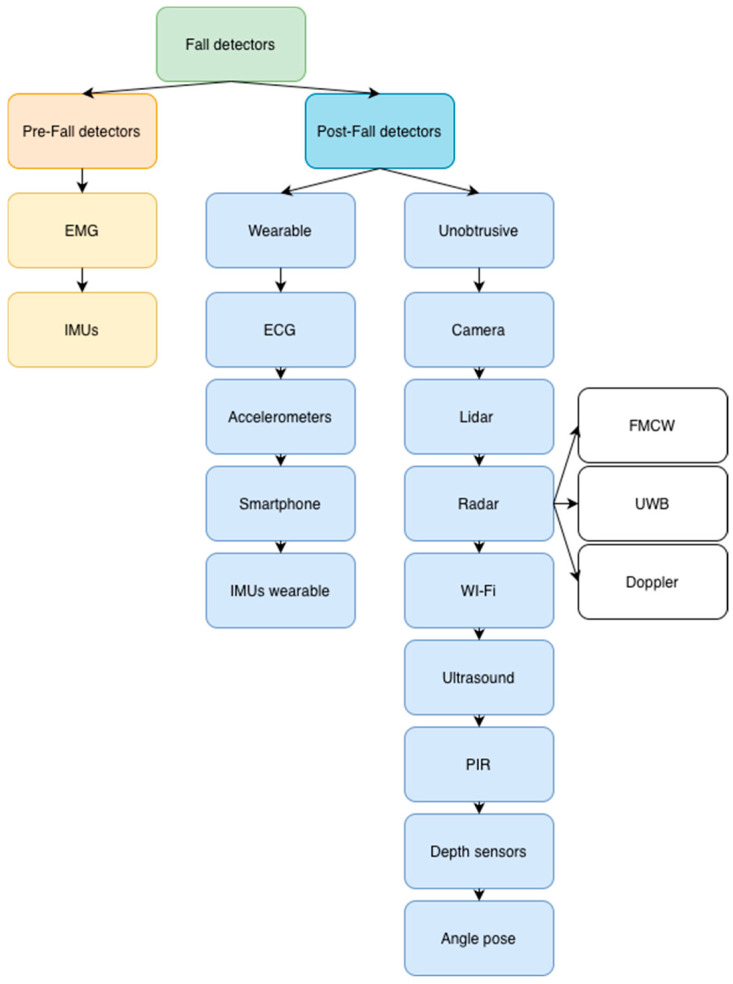
Distribution of the fall detection system. Abbreviations: EMG, electromyography; IMU, inertial measurement unit; ECG, electrocardiography; FMCW, frequency-modulated continuous wave; UWB, Ultrawide band; PIR, passive infrared.

Preventing falls is of the highest importance and should involve a multifaceted approach that includes education, training, creating safer environments, prioritizing research on falls, and establishing effective policies to reduce the risk. Falls are defined as events that occur when an individual inadvertently comes to rest on the ground, floor, or below. Although many fall-related injuries are not fatal, they can still have significant consequences [1,2,3,7,8,9,10,11]. Overall, falls are a major public health concern worldwide, with significant fatalities and non-lethal injuries. The burden of falls is particularly high in older adults and in low and middle-income countries [1,2,3,4,5,6,7,8,9,10,11]. This review aims to highlight the current state of fall research, to investigate prevention strategies and progress in the detection and prevention of falls. The review is divided as follows: First, the article provides an in-depth study of wearable sensors, and the second section describes unobtrusive sensors. Figure 1 shows the classification of fall detection methods, which are divided into fall prediction and post-fall detection.

Fall prediction is possible through EMG (electromyography) or IMUs (Inertial Measurement Units). Post-fall detection is further divided into two groups: wearable and unobtrusive devices. Following this extensive sensor analysis, the article moves into a discussion. Fall risk factors are usually divided into two groups, namely external and internal risk factors. External risk factors are those influenced by the environment. Internal risk factors are determined by patient medical history (poor vision, confusion, inability to maintain balance, etc.) [12,13]. The fall risk assessment provides an objective format for structured assessments that can be used to identify potential falls. Comprehensive fall prediction tools can help nurses identify potential threats and reduce their risk. Fall risk assessment tools are instruments used by the healthcare providers to record and evaluate risk factors. The determination of a fall prediction is an important factor in prevention. Regardless of which treatment is chosen, care should be taken to ensure that the treatment selected for the assessment of the risk of falling is sensitive and specific to the patient. Fall prevention strategies often start after the fall, not before. This is the main reason why some fall procedures cannot consistently reduce the overall incidence of fall related injuries over time [14]. Falls can be evaluated according to scales. Only six scales are mentioned in this paper, four for adult/elderly patients and two scales for assessing children. The Little Schmid or Humpty Dumpty scale is used to evaluate fall risk in pediatric patients [14,15,16,17,18,19]:

- Hendrich II fall risk model
- Morse fall scale
- St. Thomas fall risk assessment
- Schmid fall risk assessment
- Little Schmid fall risk assessment
- Humpty Dumpty

Individual rating scales indicate the number of points for a given issue, such as confusion, a history of falling, or frequent trips to the toilet, among others. Each scale has different but similar factors and different scores. These scores ultimately aim to assist healthcare professionals in assessing the risk of falling. Fall detection methods are essential for ensuring the safety and well-being of individuals, particularly the elderly and those with mobility impairments. As falls can lead to serious injuries, such as fractures and head trauma, the development of reliable fall detection systems has become a critical area of research. These systems utilize various technologies and methodologies to promptly identify falls and alert caregivers or emergency services, thereby reducing response times and potentially mitigating the consequences of such incidents [10,20,21]. In this review, we explore the various methods used for fall detection, examining the underlying technologies, methodologies, and practical applications of each. By jointly analyzing wearable and non-wearable systems across signal characteristics, commonly employed learning methods, and reported performance limitations, the review highlights recurring patterns that emerge across different technological classes. The purpose of this article is to provide a detailed overview of existing fall detection and prevention technologies, highlighting their unique features and potential for future development. In this way, we aim to offer valuable insights into the current state of fall detection research and identify promising directions for future innovation.

**Figure 2:**
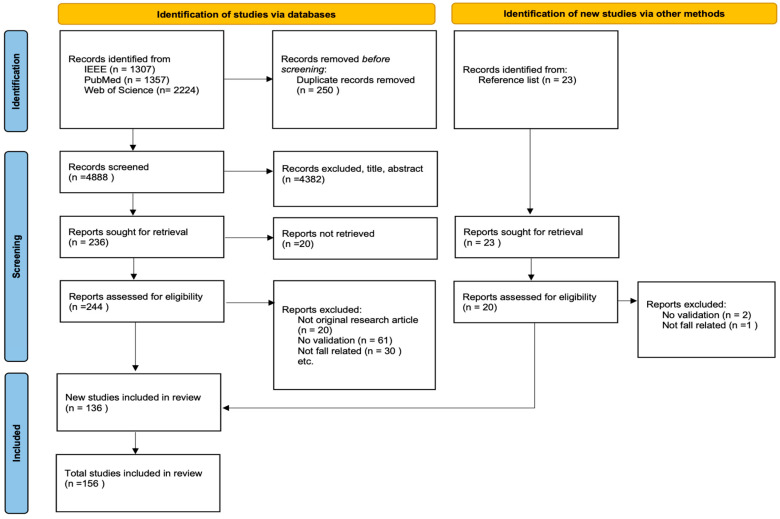
PRISMA diagram of selection process for review.

We searched IEEE Xplore, Scopus, Web of Science, and PubMed for English-language articles published between 2016 and 2025. In addition to database searching, we performed backward citation searching by screening the reference lists of relevant articles to identify further eligible studies. After exporting records from all sources, duplicates were removed. The remaining records were screened and full texts were assessed for eligibility, with reasons documented for exclusions at the full-text stage. The study selection process is summarized using a Preferred Reporting Items for Systematic Reviews and Meta-Analyses (PRISMA) flow diagram shown in Figure 2.

## 2. Pre-Fall Prediction

The aim of this chapter is to explore the concept of pre-fall detection. Fall detection before the occurrence of an actual fall is a critical aspect in healthcare settings. By identifying the potential risk of a falling before it happens, healthcare workers can proactively implement preventive measures and provide closer monitoring to ensure patient safety. In recent years, significant advances in the development of pre-fall detectors have been made, offering promising solutions for early fall detection and prevention [22].

### Detection via Biosignal and IMUs

A way to detect a fall before it occurs is to use a biological signal, namely an EMG signal. Often, electrodes are connected to leg muscles, allowing healthcare workers to assess patients’ walking patterns and therefore monitor the probability of a fall [23,24]. The EMG signal monitoring system employs a threshold-based approach that enables the detection of imbalance conditions approximately 200 ms after a disturbance stimulus under simulated and controlled fall conditions [23,24,25]. Inertial measurement units can be used together with machine learning to create and track model walking patterns over time. By tracking walking patterns and their characteristic, typical walks, abnormal walks and falls can be defined [26,27]. Disposable adhesive electrodes are not always easy to place, particularly for users without training or experience; therefore, alternative wearable solutions such as smart socks can be used. Like disposable electrodes, socks sense muscle activity from the feet and assess changes in walking [28]. Fall detection can also be performed using wearable wristbands with gyroscopes, accelerometers and EMG sensors [29,30,31].

## 3. Post Fall Detection, Wearable

This chapter explores the various technologies used in post-fall detection systems, revealing their operational principles and technological advances. Understanding the capabilities and limitations of these detectors enables healthcare professionals to make informed decisions to improve fall response and improve the overall safety of patients in healthcare environments. The rapid response to a fall is the most important factor in ensuring the safety and well-being of individuals, and the post-fall detector plays a crucial role in facilitating rapid response and assistance [2,3,4,5,6,7,8,9,10,11,12,13,14,15,16,17,18,19,20,21,22,23,24,25,26,27,28,29,30,31]. These detectors use several technologies to detect falls and trigger alert systems. One of these approaches is to use classical biological signals such as electrocardiography (ECG) to capture physiological changes associated with fall events.

### 3.1. Detection by Biosignal

By identifying the potential risks of falling before it occurs, health workers can proactively implement preventive measures and provide closer monitoring to ensure the safety of patients. One approach that has gained attention in the field of pre-fall detection is the use of classical biological signals such as electrocardiography (ECG). In a notable study by F.S. Butt et al. [32], a wearable tape with an ECG sensor connected to an Arduino was employed for fall detection. By creating a neural network to evaluate the ECG data, significant changes in signal amplitude have been observed, which could be attributed to both fall noise and an increase in heart activity [32]. In a study by R. Castalo et al. [33] an accelerometer was used in combination with an ECG device. They used an accelerometer to cut off ECG signals when changes occur, changes such as lying or standing up. This enabled the trained model to achieve high accuracy [33,34].

### 3.2. Wearables

Accelerometer-based sensors use wearable devices for detection. Three-axis accelerometers, three-axis gyroscopes and three-axis magnetometers are used to detect falls where the device is worn on the patient’s waist [35] or ankle [36,37]. The accelerometer records acceleration data at rate of 50 Hz. Data collection is carried out in real time and information is processed by the microcontroller. The accelerometer-based data can be transmitted via Bluetooth Low Energy (BLE) to a computer monitoring application. This allows data visualization and storage in real time. To label the collected data, synchronized video recordings can be used. A webcam captures the user’s activities while collecting accelerometer data. Each segment is labeled using the corresponding video recording and classified by a neural network as background, normal movement, fall or risk [2,10,36,38]. The accelerometer-based fall detection system can predict an impending fall at least 70 ms in advance and provides kinematic measurements related to body motion and orientation [39,40]. This sensor is strategically located near the center of gravity to improve the accuracy of fall detection. The algorithm used for fall detection relies on a threshold-based approach. The main variable taken into account in this approach is the vertical velocity of the inertial frame. By monitoring this variable and applying a threshold, the system aims to distinguish between non-fall activities and fall events [38,41,42]. Another approach is to use a near-fall detection system using Hidden Markov Models with data from multiple Inertial Measurement Units (IMUs) placed on the user’s body, including the torso and thighs. Therefore, it should be possible to detect near-fall events before they cause injuries [39,40,43,44].

Wearable devices that detect falls are most often attached to the waist with a device. With the help of three-axis accelerometers, three-axis gyroscopes, three-axis magnetometers, and barometer sensors integrated into the device, a very accurate estimate of the position and height of the patient can be obtained, and a fall is decided using a preset threshold [45]. Wearable devices enable low power consumption and efficient timing signal analysis and also continuous monitoring, reducing device size and increasing battery life. As a disadvantage, these devices must be charged regularly and worn by the patient. Deep learning models based on recurrent neural networks can be used to detect falls in real time with accelerometer-based signals. The principle of deep learning for fall detection involves using multiple layers of neurons to process raw input data [2,3,8,46].

### 3.3. Smartphone

Fall detection with sensor technology in mobile phones offers well-tested and widely available communication services. The data recorded by accelerometers and gyroscope sensors automatically evaluate the fall when a threshold is set to a specific threshold according to predefined parameters or with the help of machine learning. Several fall detection systems using phones already exist, each using a different mobile phone with different integrated sensors [2,26,47,48]. A three-axis wearable accelerometer was used as a reference [49].

## 4. Post Fall Detection, Unobtrusive

In recent years, the field of post-fall detection has made significant progress thanks to the advent of non-obtrusive sensor technologies. The chapter explores the fascinating field of unobtrusive sensors post-fall detection, which plays a key role in ensuring the safety and well-being of individuals, especially in health and elderly care settings. These sensors are available in a variety of forms, each with unique capabilities to detect falls events and potentially life-threatening situations. Among the various sensor technologies, camera-based detection systems analyze visual data using computer vision techniques and identify falling patterns. In addition, LiDAR, radar, depth sensors, and ultrasound technology can detect environmental changes and human presence changes that indicate falling. Wireless signals, including Wi-Fi and smartphone connections, are used to identify falls by reviewing interference or signal strength fluctuations. In addition, passive infrared (PIR) sensors, temperature sensors and vibration detectors provide valuable insights into sudden movements or abnormal patterns that may indicate falls.

### 4.1. Camera Based

For the fall detection based on cameras, there may be an issue with sensitive data. Camera-based fall detection can isolate the person from the background using the deep learning model and follow them. Through the adaptation of the neural network, it changes dynamically to match the current visual characteristics of the environment. As a result, object classification and tracking accuracy is guaranteed. The video is processed to isolate human figures from the background, and key features of their movements are extracted using techniques like Convolutional Neural Networks (CNNs) [50,51]. These features are then analyzed over time with Recurrent Neural Networks (RNNs) or Long Short-Term Memory (LSTM) networks to detect abnormal movements indicative of a fall. In order to increase classification efficiency, semi-supervised learning strategies are used [51,52,53,54,55]. Another approach involves the use of the wireless sensor network (WSN) combined with the k-nearest neighbor algorithm. In this case, falls are detected by identifying characteristic fall pattern within the data stream [38,51,56].

### 4.2. LiDAR

LiDAR is a sensing technology that uses laser light to measure distance and create high-resolution maps or 3D models of objects, as well as to accurately monitor and detect falls in fall detection systems. When these laser pulses meet objects in their field of view, including a person, they bounce back towards the sensor. The sensor consists of components such as photo-current generation photodiodes on the chip, a transimpedance amplifier for converting photo-current to voltage, a post amplifier and a digital time converter for estimating the target distance [57]. This data helps to determine the distance between the sensor and the object from which the signal is reflected, such as the person. To detect a fall, the algorithm considers various parameters, including the existence of a person and their movement patterns. When the algorithm determines a significant change in the person’s presence or detects an abnormal pattern of movement (a signal of falling), an alarm or alert is triggered. This makes it suitable for falling detection by analyzing changes in the presence of a person and movement patterns [58,59] like walking, sitting, standing, falling [60]. The LiDAR system includes a neural processing unit for motion detection and decision-making [57]. The system’s ability to operate in areas such as bathrooms is an important feature because it is not based on cameras, protecting privacy while still providing essential monitoring and safety benefits [58].

### 4.3. Radar

Radar technology provides fall detection that ensures privacy. The radar system emits radio waves (microwaves or radio frequency signals) and measures the time these waves take to bounce back after hitting an object [1,61,62]. The millimeter-wave-based sensors operate in the frequency range of 60–64 GHz and provide cloud data points that are usually analyzed with various machine learning and deep learning algorithms [8,26,27,28,29,30,31,32,33,34,35,36,37,38,39,40,41,42,43,44,45,46,47,48,49,50,51,52,53,54,55,56,57,58,59,60,61,62,63]. The wavelength of radar electromagnetic waves in this frequency range is about 5 mm [8,26,27,28,29,30,31,32,33,34,35,36,37,38,39,40,41,42,43,44,45,46,47,48,49,50,51,52,53,54,55,56,57,58,59,60,61,62,63]. The radar system emits continuous signals or pulses. Radars can detect not only stationary objects but also moving ones. When a person is in the radar field and starts moving or falling, their body reflects the radar wave. The radar system continuously measures the change in the round-trip time of the wave reflected from the moving person. These data are then processed using advanced algorithms to analyze the motion patterns. By distinguishing between normal activities and the rapid changes in movement associated with falls, the system can accurately detect when a fall occurs [61,63,64].

#### 4.3.1. Doppler Radar

The Doppler radar is used to detect falls by monitoring changes in the Doppler frequencies of radar signals that are reflected from the body of a person. Doppler radar systems emit continuous or pulse radio waves in the surrounding environment at 5.8 GHz [64,65]. These radar waves move at the speed of light. As a person moves, their body moves, and the frequency of the radar waves reflects these changes. This frequency change is called the Doppler effect. When a person is stationary, the reflected radar waves have a constant frequency. However, when people move, the frequency of the reflected waves changes [1,65,66,67]. The radar signature obtained from the reflection wave is calculated in a spectrogram, then the energy burst curve is calculated on the basis of the spectrogram. This curve summarizes the energy levels in a specific frequency range of interest for fall detection. Falls are considered to be events in which several parts of the body move at relatively high speeds and result in higher energy levels at various frequencies [67,68].

Radar systems use time-frequency analysis techniques to study the Doppler frequencies of the reflected signals over time. This analysis helps identify patterns and movements. In order for fall detection to be effective, the radar system must establish a baseline for the normal movement patterns of the person. This baseline helps the system distinguish between regular activities (e.g., walking or sitting) and fall events. The detection algorithm, often implemented using machine learning and deep learning techniques [55,64,65], or wavelet transform [65]. The fall detection algorithm is usually designed to trigger alerts when certain thresholds or predefined criteria are met. These criteria are based on the rate and magnitude of Doppler frequency changes that indicate a sudden and abnormal movement change consistent with a fall [67,68,69]. When the algorithm detects a fall-like pattern or meets the predefined criteria, it triggers an alert or notification [66].

#### 4.3.2. Frequency-Modulated Continuous Wave (FMCW)

FMCW radar transmits a frequency-modulated signal, by comparing the frequency of the transmitted signal with the frequency of the received signal, the radar can determine the time delay, and hence the range, to the target. FMCW makes it possible to distinguish fall detection from movements in everyday life. In a study by Ding Ch. A et al. [70] who propose a Dynamic Range-Doppler Trajectory method, they use the FMCW radar system to extract multi-domain features extracted from the reflected signals. The features are then used for K-nearest neighbor (KNN) machine learning. The radar in this case operates at a frequency of around 5.8 GHz. The generated signal is divided into two parts using a power divider, where one part is used as a reference signal while the other is transmitted through the antenna. The signal reflected from the object is then received by the receiving antenna [70,71].

In study by Yao Y. et al. [72], the radar signal undergoes Fast Fourier Transform (FFT) to extract range and velocity information, with static object removal to isolate moving targets. The system features a 3-D convolutional autoencoder (ResNet-based) to extract motion features and a Deep Neural Network (DNN) predictor to learn non-fall action patterns [72,73]. In study by Liang T. et al. [74] a system consisting of three main components were used: point cloud enhancement (PCE) for improving point cloud quality, a feature extractor for extracting human pose parameters, and a classifier for distinguishing between normal and fall events. The PCE model transforms low-quality point clouds into high-quality ones by reconstructing their shapes using a novel 3D point-box hybrid regression loss function [74]. Expanding on potential applications of FMCW radar for fall detection, the system can utilize a 2D Fourier Transform to generate range-velocity maps, enabling the capture of intricate motion patterns. It also introduces innovative features like centroid range and range width to effectively distinguish falls from other motions [75].

#### 4.3.3. Continuous Wave

Monostatic Continuous Wave (CW) radar systems analyze the reflection of continuously transmitted electromagnetic waves that return from objects such as humans. When these encounter a human body, they scatter and reflect back to the radar receiver, and the characteristics of the returned signal depend on the motion and physical properties of the person. CW radar inherently measures the Doppler shift caused by the relative motion between the radar and a target, which allows velocity information to be inferred from the frequency shift of the returned signal [68,76,77]. In order to detect falls and other human activities, radar returns are analyzed in the time-frequency domain (TF). This analysis involves techniques such as short-time Fourier transformation (STFT) to break down non-stationary Doppler and microDoppler signals generated by human movements. The STFT provides a spectrogram showing how the signal power changes over time and frequency [76,77,78]. Specific features, such as fall, can be extracted from the spectrogram. A classification algorithm, often a support vector machine (SVM), is applied to classify the human activity based on the extracted features. For fall detection, the classifier distinguishes between fall events and other activities like sitting, bending, straightening, or walking [76,77,79].

#### 4.3.4. Ultrawide Band (UWB)

In the study by Diraco G. et al. [61], fall detection is performed by a combination of micro-Doppler features extracted from radar signals and event detection methodology. First, the radar-derived signal is filtered and modified to ensure further processing. This involves the use of filters to remove noise and normalize the data. Micro-Doppler features are then extracted from the preprocessed signal. These features originate from the micro-motions of various human body parts occurring during dynamic actions, such as falling. To extract the micro-Doppler features, Doppler shift spectroscopy is employed to characterize the signal’s power distribution as a function of velocity and range. This method is based on the principle of comparison between micro-Doppler features acquired during activities of daily living (ADL) and those acquired during fall. The fall is then detected as an “event” or deviation from the usual activities. For a more accurate detection, simple K-means classifiers are used to vote on whether an event matches normal activities [61,80,81].

### 4.4. Ultrasonic Sensor

Ultrasonic sensors operate on the basis of the flight time principle, which emits a pulse signal to an object and then measures the time it takes for the reflected signal to return. This time measurement is used to calculate the distance to the object. The distance (d) is calculated using the formula d = c_air × t/2, where c_air is the speed of sound in air, and t is the measured time. Ultrasonic sensors generally use an approximate frequency of 40 kHz and an 8-pulse signal waveform. The maximum distance that these sensors can measure is about 254 inches. The width of the return sound beam is used to determine the position of the person when compared to the default scenario (empty room). A rapid transition from standing position to sitting position is classified as a fall. To enhance accuracy, the system continuously monitors the changes in the distance measurements and the speed at which these changes occur. When a sudden and significant drop in height is detected, along with a corresponding shift in the return sound beam’s width, the sensor interprets this as a fall [1,2,3,4,5,6,7,8,9,10,11,12,13,14,15,16,17,18,19,20,21,22,23,24,25,26,27,28,29,30,31,32,33,34,35,36,37,38,39,40,41,42,43,44,45,46,47,48,49,50,51,52,53,54,55,56,57,58,59,60,61,62,63,64,65,66,67,68,69,70,71,72,73,74,75,76,77,78,79,80,81,82].

### 4.5. Wi-Fi Detection

The introduction of fall detection by Wi-Fi technology represents a groundbreaking advancement in elderly care, enabling real-time monitoring and rapid response to potential accidents, ultimately enhancing the safety and well-being of seniors living independently. The wearable fall detection device is based on the acquisition of Wi-Fi Channel State Information (CSI) [83]. The fall detection solution is based on time-frequency analysis, which is also used in radar fall detection. STFT (conventional short-time Fourier transform) is used to extract time-frequency features along with a sequential forward feature selection algorithm to select features robust to environmental changes [84,85]. In the paper by Y. Wang et al., a correlation between different radio signals and radio propagation model analysis was investigated. Based on the observations, the WiFall was proposed. It is a fall detection system that utilizes a CSI -based activity indicator [83]. WiFall can detect falls without requiring any hardware modifications or the use of wearable devices. The system was implemented on a desktop computer and demonstrated high accuracy in detecting the fall of a single individual [83,84,85,86]. Technology system with deep neural architectures enables continuous, unobtrusive monitoring for seniors living independently, providing rapid alerts that can dramatically improve emergency response and overall well-being [85].

### 4.6. Depth Sensor

Depth sensors emit infrared patterns and compute a per-pixel distance map at ≈30 fps, producing synchronized RGB-depth (RGB-D) frames. This 3-D information enables robust pose estimation even in low-light or completely dark environments because the infrared signal is independent of visible illumination [87,88]. Fall detection uses a fuzzy inference system, whereas the disadvantage of this system is that tracking is possible only in limited areas. To complement areas that are not recognized by the camera, wearable motion detection devices such as accelerometers and gyroscopes can be added [88,89]. The Kinect is a depth-sensing camera originally designed for gaming that has become a popular low-cost platform for monitoring human motion in healthcare applications. It emits an infrared (IR) pattern (structured-light projector in Kinect v1, time-of-flight sensor in Kinect v2) and records the reflected pattern with an IR camera. By triangulating the deformation of the pattern (or measuring the round-trip time of IR pulses) [88]. From the depth map, the built-in software development kit extracts a 3-D skeletal model (≈25 joints) using machine-learning–based body-part classification. Fall detection algorithms then monitor joint trajectories, velocities, and postural angles (e.g., rapid increase in torso-to-ground distance followed by a sudden vertical drop) [90]. When a potential fall is detected, typically indicated by unusual acceleration, the system processes the depth data to confirm the event. Algorithms analyze features such as body orientation and velocity to distinguish falls from other activities [86,88,89,91].

### 4.7. InfraRed Camera Based

Infrared cameras can be used as fall detectors (thermal/infrared cameras). The sensor outputs were converted to images and supervised deep learning was applied to the images. The deep learning network included convolutional neural networks for automatically extracting features from infrared images [92,93]. In an article by J. Nogas et al. [94] the DeepFall system was proposed to test publicly available sensing modalities, namely thermal cameras and depth cameras. The system uses deep spatial-temporal convolutional autoencoders to determine spatial and temporal characteristics of common activities to detect falls. A depth-autoencoder that ignores 2D image structures is also used to find features of everyday life activities [94].

### 4.8. Acoustic

Using three antenna microphones and a floor acoustic sensor, acoustic information can be monitored in the room. The data obtained from the microphone and floor sensor are used to infer whether a fall occurred. The acoustic characteristics (energy, spectral centroid, spectral flow, transmission rate, and frequency cepstral coefficients) are extracted from the acoustic signal [95,96]. The data can be processed by support vector machines (SVM) or deep learning neural network with a reduced feature set obtained by principal component analysis (PCA) from the acoustic features. These characteristics help in identifying and distinguishing fall events from other sounds in the environment [95,96].

### 4.9. Vibration

In general, vibration-based fall detection systems can be implemented using the floor. Vibrations from footsteps and falls are analyzed. It is possible to work in real time, allowing the system to detect a fall immediately and identify a person based on one or two footsteps. A method of collaborative network location is used, in which sensors work together to recognize a person if they move or if a fall has occurred. The state of walking can be estimated by means of sound source estimation methods with multiple microphone sensors. When a fall occurs, it generates distinct vibration patterns or sudden spikes in the sensor data. The system processes these vibrations to identify characteristic fall signatures, distinguishing them from normal activity or other disturbances [97,98,99,100].

### 4.10. PIR Sensors

Passive infrared (PIR) sensors offer the great advantage of being placed in bathrooms where falls are very common. The sensor is then connected to a device to send information to the family or medical staff about the patient’s condition [101]. PIR sensors are passive devices that can detect the presence of objects (including humans) based on the heat (infrared radiation) they emit [102,103]. They are composed of two pyroelectric sensors placed side by side. When objects, such as humans, move in the sensor field of view, the temperature difference between objects and their surroundings causes the sensor to recognize these changes in infrared radiation. The fall processing and evaluation algorithm extracts statistical characteristics such as average, area temperature and duration, as well as fall symptoms. A neural network is used to distinguish between a fall and a normal non-fall event [103,104,105]. Each sensor is characterized by a specific field of view and a corresponding detection area. In the context of the detection of falls, these sensors are usually mounted on walls near the patient’s bed and are used to cover the area where a fall can occur. When fall occurs or the patient makes a sudden and significant movement, its body temperature change triggers the PIR sensor [101]. This change in infrared radiation is detected by the PIR sensor as a high-intensity signal. To distinguish falls from regular movements, the system analyzes the signal properties. Falls generally involve sudden and fast movements, which leads to a distinctive pattern in the output signal of the PIR sensor. The system compares the signal intensity, speed and other characteristics with predefined thresholds and patterns to identify fall events [101].

### 4.11. Angle Pose System

With the help of OpenPose, it is possible to obtain information about the skeleton of the human body and to identify the fall using three parameters. The rate of descent at the center of the hip joint, the angles of the axis of the human body with the ground and the height of the human body, indicate whether or not a fall is occurring [106,107].

## 5. Discussion

Taken together, current evidence indicates that the main bottleneck in fall monitoring is no longer the availability of sensing modalities, but the practical balance between sensitivity, robustness, and deployability in real care environments. Pre-fall detection is conceptually attractive because it targets prevention rather than reaction; nonetheless, its reliability depends on capturing subtle, person-specific deviations in gait and neuromuscular control. In this context, combining EMG with IMUs is frequently presented as a stronger strategy than either modality alone, yet the clinical value is tempered by operational burdens such as electrode placement, recurring maintenance, and cost, which can reduce long-term adherence outside controlled studies. Post-fall detection via wearables and smartphones remains comparatively mature and scalable, but performance reported in laboratory datasets may not translate directly to routine practice when battery constraints, inconsistent wearing behavior, and heterogeneous movement patterns are considered. Unobtrusive sensing addresses compliance and enables continuous observation, but shifts the challenges to privacy, coverage, and environmental variability; camera-based pipelines can achieve high precision with modern pose-estimation models, yet their acceptability and reliability in low-light settings remain critical limitations. Privacy-preserving alternatives (e.g., radar, LiDAR, Wi-Fi, acoustic, vibration, PIR, and depth sensing) broaden deployment options, but often require careful calibration and context-aware modeling to manage interference and reduce false alarms, suggesting that successful implementations will likely rely on adaptive fusion and clinically meaningful validation rather than single-modality benchmarks. Table 1 provides a brief comparison of the discussed methods, and Table 2 summarizes their limitations.

### 5.1. Algorithmic Trajectory Beyond Sensors

Across sensing modalities, the reviewed fall-detection literature follows a converging algorithmic trajectory that is not captured by sensor-based taxonomies alone. Early and resource-efficient systems typically rely on thresholding and rule-based triggers (e.g., inertial sensing or ultrasound height-change heuristics), which are attractive for real-time operation but are strongly sensitive to calibration, device placement, and user compliance. A second wave replaces fixed rules with classical machine-learning pipelines: domain experts design time- or frequency-domain descriptors (including time–frequency representations such as spectrograms), followed by conventional classifiers such as k-NN or SVM. Notably, this pattern appears across domains—radar and Wi-Fi both exploit time–frequency analysis, while several radar studies further combine engineered multi-domain features with k-NN or event-detection strategies. More recent work increasingly shifts toward representation learning, where models learn discriminative motion or pose representations directly from raw or minimally processed inputs. Examples include temporal deep networks for accelerometer streams, CNN/RNN hybrids for video-based analysis, and radar pipelines that learn motion features via autoencoders or improve sensing fidelity through point-cloud enhancement before classification. In vision-based approaches, algorithmic progress is also driven by stronger structural priors: skeleton-cue encodings, limb-direction modeling, and transformer formulations that capture global bone–point relations, illustrating how core concepts evolve from appearance-based cues to explicit geometry and relational reasoning.

Advanced deep learning architectures such as Transformers, foundation models, and large language model (LLM)-inspired networks are indeed emerging for time-series and spatio-temporal data analysis, including fall detection. These models leverage self-attention to capture complex sequential patterns and long-range temporal dependencies, which can improve recognition of falls and activities [115,116]. For example, transformer-based approaches in wearable-sensor fall detection have outperformed traditional CNN/LSTM models, achieving higher accuracy and reducing false alarms by better modeling subtle motion differences [116,117]. The potential benefits—improved sequence modeling, multi-sensor integration, and rapid adaptation to new conditions—suggest that Transformers and other foundation model approaches could significantly advance fall detection capabilities as this research area evolves.

### 5.2. Human Activity Recognition

Human activity recognition (HAR) is the computational task of automatically identifying human actions from raw sensor data, enabling machines to understand and react to everyday moments [118]. HAR draws on a wide variety of modalities, including RGB video, depth or infrared imaging, skeletal data, and, most prominently, inertial measurement units (IMUs) that capture acceleration, angular velocity, and magnetic fields [119,120]. Wearable IMUs placed on the waist, wrist, or neck provide continuous kinematic streams that, when processed with threshold-based, support-vector-machine, or deep-learning classifiers, can alert caregivers in real time [111,112,113,114,115,116,117,118,119,120,121]. In traditional HAR, each window is passed to a feature-extraction stage where domain experts craft statistical, frequency-domain, or biomechanical descriptors (hand-crafted features) [122]. These features are fed to conventional machine-learning classifiers—k-NN, SVM, Random Forest, etc.—that are trained on annotated examples [123]. Overall, HAR’s principle is to convert multimodal sensor streams into informative representations—either engineered or learned—and apply supervised learning to map them to activity labels, enabling applications in health monitoring, smart environments, and beyond. From this perspective, fall detection can be considered a specialized subset of human activity recognition that focuses on special events. The sensing modalities and processing pipelines reviewed for fall detection—particularly IMU-based motion analysis and temporal deep-learning models—are largely transferable to broader HAR tasks such as daily activity classification or posture transitions [108,109,110,111,112,113,114,115,116,117,118,119,120,121,122,123,124,125,126,127,128,129,130].

**Table 2:** Comparison of limitation across methods. Abbreviations: SVM, support vector machine; PCA, principal component analysis; CSI, channel state information; LSTM, long short-term memory; GRU, gated recurrent unit; DeFall and SIFall denote method names reported in the referenced studies.

Technology	Usage	Limitation	Precision	References
Inertial measurement units (IMUs)	Pre-fall detection	Sensor placement strongly influence outcome High false alarm rates in real world use Algorithmic generalization Latency—detection before fall User comfort—wearing multiple units, discomfort	Reduced precision (drop to ≈60% when applied on actual falls) Wide specificity variation (≈83% ± 30%)	[108,109,131,132]
Biosignal	Pre-fall detection	Quality may vary widely across subjects (weight, height, another anthropometric) Tight skin contact or multiple sites High false detection	Sensitivity ≈ 19%	[24,25,29,131]
Camera	Post-Fall detection, unobtrusive	Light (no/visibility) Multiple occupants Privacy-preserving	Precision typically 70–80%	
		High cost		[56,57,58,59,60,61,62,63,64,65,66,67,68,69,70,71,72,73,74,75,76,77,78,79,80,81,82,83,84,85,86,87,88,89,90,91,92,93,94,95,96,97,98,99,100,101,102,103,104,105,106,107,122,124,125,126,133]
		False alarms—careful calibration and optimization		
Biosignal	Post-Fall detection, wearable	Skin irritation, differences in body shape	Real world precision 57–80%	[29,32,132]
Wearables	Post-Fall detection, wearable	Skin irritation, skin contacts, sensor placement, often occurring of false alarms, lack of real world datasets	In laboratory tests precision ≈ 90%	[29,109,123,131]
Smartphone	Post-Fall detection, wearable	Skin irritation, privacy concerns, miss low impact falls	Precision range ≈ 60–90%	[48,134,135]
Inertial measurement units (IMUs) wearable	Post-Fall detection, wearable	Skin irritation, sensor placement, high false positive rates	Good results in laboratory environment (90% precision), in real world ≈ 60%	[124,125,136]
Lidar	Post-Fall detection, unobtrusive	Limited range, environmental sensitivity	2D-lidar 98% combined with CNNs	[57,58,60]
Radar	Post-Fall detection, unobtrusive	Reflective surface, performance varies markedly with radar height	Precision ≈ 90%, multiple LiDAR increase precision (98%)	[68,76,77,137]
Ultrasonic sensor	Post-Fall detection, unobtrusive	Objects that are close together can be indistinguishable, environmental sensitivity Objects closer than a few centimeters cannot be measured	Accuracy ≈ 90–98%	[114,137,138]
Wi-Fi	Post-Fall detection, unobtrusive	Antenna orientation Occlusion and blockage Signal ambiguity	DeFall 95% SIFall 98% accuracy Deep learning CSI systems reach 96%	[83,85,139,140]
Depth sensor	Post-Fall detection, unobtrusive	Occlusion—inaccurate depth Accuracy degrades beyond ≈4.5 m Single person tracking	Typical precision of ±1–3 mm within its optimal range (0.5–4.5 m for v2) Accuracy above 90% in laboratory studies	[88,90,141]
InfraRed camera	Post-Fall detection, unobtrusive	Occlusion Light, Similar temperature object	95% accuracy With use of CNNs 96%	[93,102,142]
Acoustic	Post-Fall detection, unobtrusive	Ambient sound interference Walls, furniture or a person’s body can block or attenuate the wave, precision is bounded by ambient noise	Precision reaches ≈ 86% SVM classifiers on PCA-reduced acoustic features typically achieve 80–90% accuracy, but sensitivity can drop below 70% in noisy rooms	[29,58,60,96,97,98,99,100,101,102,103,104,105,106,107,108,109,110,111,112,113,114,115,116,117,118,119,120,121,122,123,124,125,126,127,128,129,130,131,132,133,134,135,136,137,138,139,140,141,142,143]
Vibration	Post-Fall detection, unobtrusive	Occlusion Environmental noise (footsteps, dropping object)	90% accuracy Combination with k-means 100%	[97,100,144]
Passive infrared (PIR) sensor	Post-Fall detection, unobtrusive	High temperature, pets can activate, multiple people in room, Sensor detect just movement	In controlled lab test 75–85% precision Use of two sensors ≈95% sensitivity	[101,102,104,105,113]
Angle pose system	Post-Fall detection, unobtrusive	Occlusion Light, visual noise Misclassifying rapid sitting	OpenPose + LSTM/GRU 98%	[106,145,146]

Table 2 highlights that, across sensing modalities, the dominant bottleneck is not achieving high performance under controlled conditions but maintaining robustness and generalization in real-world deployments. A recurring pattern is the pronounced performance drop outside laboratory settings, driven by domain shift (differences in subjects, fall types, and activities of daily living), deployment variability (sensor placement and orientation), and environmental confounders.

For wearable IMU-based approaches, Table 2 indicates that strong laboratory results do not consistently translate to practice, where studies report substantially reduced accuracy and highly variable specificity. This suggests that model decision boundaries are frequently entangled with subject-specific movement patterns and with ADL events that mimic falls (rapid sitting, kneeling, lying down, abrupt turns). Importantly, wearables also face adoption barriers—compliance, comfort, and battery dependence—which can introduce missing data and decrease long-term reliability. From a deployment perspective, these limitations imply that reporting only accuracy is insufficient; studies should emphasize false-alarm rate (e.g., false alarms/day), robustness to unconstrained ADL, and performance stratified by subject and context.

For biosignal-based methods (e.g., EMG/ECG), Table 2 underscores two central constraints: strong inter-subject variability and dependence on signal quality/contact conditions, and limited standalone robustness for fall-related dynamics, particularly when distinguishing fall-like transitions from benign physiological fluctuations. This makes biosignals more suitable as complementary inputs (e.g., for decision refinement or confirmation) rather than universal single-sensor solutions, especially in unconstrained environments. Among unobtrusive modalities, Table 2 reveals a clear trade-off between privacy, robustness, and practical feasibility. Vision-based solutions can achieve good performance, but are constrained by privacy concerns, sensitivity to illumination, and susceptibility to occlusion and multi-person interference. Depth/IR mitigates some lighting issues but still suffers from occlusion and distance-related degradation. LiDAR often reaches high reported accuracy in controlled experiments yet is limited by cost and installation constraints. In contrast, radar stands out as a privacy-preserving option with strong potential for in-home use, while Table 2 makes explicit that radar performance can be strongly affected by installation geometry (height, angle), multipath reflections, and environmental clutter.

Several low-cost alternatives (e.g., ultrasonic sensing, Wi-Fi/CSI-based methods, PIR, acoustics, vibration sensors) can yield impressive metrics in selective scenarios, but their limitations tend to be deployment-critical: signal blockage, orientation sensitivity, ambiguous signatures, and strong dependence on environmental conditions. In practice, these systems can be prone to confusion (e.g., falls vs. dropped objects, footsteps, pets, or furniture interaction).

Overall, Table 2 supports the conclusion that no single modality simultaneously optimizes accuracy, privacy, robustness, cost, and user acceptability. These observations naturally point to future directions with higher translational value: multimodal fusion (to reduce ambiguity and false alarms), domain adaptation/personalization (to address inter-subject and inter-environment variability), and standardized evaluation frameworks that consistently include latency, false alarms/day, multi-person scenarios, and clinically relevant confounders such as fall and lying/rest transitions.

### 5.3. Impact of ML Model Hyperparameters on Fall Detection Performance

Threshold-based fall detection methods are very sensitive to the chosen threshold value. A tight threshold can reduce false alarms but at the cost of missed falls, whereas a looser threshold catches more falls but generates more false alerts. Thresholds do not generalize well, using higher thresholds to improve specificity often misses subtler falls, whereas lower thresholds improve sensitivity at the expense of more false alarms. Fine-tuning threshold hyperparameters is crucial, and can secure 96% sensitivity [147,148].

Compared to static thresholds, machine learning (ML) models (e.g., SVMs, CNNs) generally achieve higher accuracy and better generalization, but their performance still depends on model hyperparameters [147,149,150]. Studies have shown that even basic ML models outperform threshold rules in fall detection. For instance, an Internet of Things (IoT) fall detector optimized with ML achieved ~90–95% sensitivity using a Support Vector Machine (SVM) or k-NN classifier, whereas a tuned threshold method yielded noticeably lower precision and adaptability [147,148,149,150]. For example, threshold-based detectors often falter on varied motions (e.g., quick sit-to-stand) that trigger false alarms, whereas data-driven models can learn to distinguish such patterns [56,147,148,149,150].

Deeper neural networks (CNNs) can capture more complex fall features, but their architecture hyperparameters (e.g., number of layers, neurons per layer) critically influence performance. In the context of fall detection, this means an overly complex CNN might perfectly classify falls in lab data but misclassify unusual activities as falls in real life (raising false alarms). One analysis notes that expanding a model’s complexity (adding layers or a huge reservoir) improves its fit on training data but can severely harm generalization, causing erratic performance on unseen activities [56,149,150,151].

A key hyperparameter for Echo state networks (ESNs) is the reservoir size (number of recurrent nodes), which strongly affects the model’s memory capacity and pattern recognition ability. ESN performance is highly sensitive to its hyperparameters. For instance, increasing the reservoir size tends to boost an ESN’s accuracy by improving its nonlinear mapping capability, but if the reservoir is too large it may overfit noise, harming generalization [151].

Across all fall detection modalities—wearable sensors (accelerometers, gyroscopes, EMG), prefall predictors, and ambient/unobtrusive sensors (camera vision, radar, depth, etc.)—there is a notable gap in the literature regarding hyperparameter sensitivity analyses. Most studies introduce a single model with a fixed configuration and report its accuracy on a given dataset.

## 6. Conclusions

This review surveyed the current landscape of fall-detection and fall-prevention technologies, contrasting wearable solutions (IMUs, EMG, ECG, smartphone-based systems) with a broad set of unobtrusive modalities (camera/RGB-D, LiDAR, radar, FMCW/UWB, Wi-Fi/CSI, thermal, acoustic, vibration and PIR systems). Across modalities, laboratory performance is often encouraging, but real-world deployment repeatedly exposes gaps: high false alarm rates, limited generalization across subjects and environments, occlusion and multi-occupant challenges, privacy and acceptability concerns, and a shortage of representative, annotated real world datasets. Importantly, no single sensor class uniformly solves accuracy, privacy, robustness and cost. Instead, complementary fusions and system level design choices appear to offer the most practical path forward.

In summary, the most impactful near term progress will come from studies that combine multimodal sensing chosen to satisfy privacy/cost constraints, rigorous real world validation and standardized reporting of operational metrics, and attention to human factors, explainability and deployment pathways. If researchers and funders reorient toward standardized datasets, prospective trials, privacy first approaches, and reproducible evaluation, fall detection systems will more rapidly mature from promising prototypes to reliable, accepted tools that reduce injury and improve outcomes for older adults and vulnerable populations.

## Figures and Tables

**Table 1:** Brief comparison of methods.

Methods	Pros	Limitations	References
Pre-fall detection	Creating models of typical walking patterns and identifying deviations indicative of a potential fall	Require careful calibration, Sensor placement strongly influence outcome	[108,109,110]
Post-Fall Detection (Wearable)	Real-time monitoring and fall detection	Battery life and patient compliance Sensor placement-affect precision	[111,112]
Post-Fall Detection (Unobtrusive)	Privacy-preserving monitoring	Limited range Dependence on fixed sensor placement	[60,75,76,88,113,114]
		Struggle to distinguish between lying due fall and sleep/rest/exercise	
		False alarms—careful calibration and optimization Multiple occupants in room	

## Data Availability

No new data were created or analyzed in this study.

## References

[1] Singh A., Rehman S.U., Yongchareon S., Chong P.H.J. (2020). Sensor Technologies for Fall Detection Systems: A Review. IEEE Sens. J..

[2] Igual R., Medrano C., Plaza I. (2013). Challenges, issues and trends in fall detection systems. Biomed. Eng. OnLine.

[3] Tanwar R., Nandal N., Zamani M., Manaf A.A. (2022). Pathway of Trends and Technologies in Fall Detection: A Systematic Review. Healthcare.

[4] Qiu H., Rehman R.Z.U., Yu X., Xiong S. (2018). Application of Wearable Inertial Sensors and A New Test Battery for Distinguishing Retrospective Fallers from Non-fallers among Community-dwelling Older People. Sci. Rep..

[5] Sczuka K.S., Schwickert L., Becker C., Klenk J. (2020). Re-Enactment as a Method to Reproduce Real-World Fall Events Using Inertial Sensor Data: Development and Usability Study. J. Med. Internet Res..

[6] Greene B.R., McManus K., Redmond S.J., Caulfield B., Quinn C.C. (2019). Digital assessment of falls risk, frailty, and mobility impairment using wearable sensors. npj Digit. Med..

[7] Merrett G.V., Tan Y.K., Merrett G.V., Tan Y.K. (2010). Wireless Sensor Networks: Application—Centric Design. https://www.intechopen.com/books/135.

[8] Khan S.S., Hoey J. (2017). Review of fall detection techniques: A data availability perspective. Med. Eng. Phys..

[9] Delahoz Y.S., Labrador M.A., Delahoz Y.S., Labrador M.A. (2014). Survey on Fall Detection and Fall Prevention Using Wearable and External Sensors. Sensors.

[10] Nooruddin S., Islam M.M., Sharna F.A., Alhetari H., Kabir M.N. (2022). Sensor-based fall detection systems: A review. J. Ambient Intell. Humaniz. Comput..

[11] El-Bendary N., Tan Q., Pivot F.C., Lam A. (2013). Fall Detection and Prevention for the Elderly: A Review of Trends and Challenges. Int. J. Smart Sens. Intell. Syst..

[12] Horová J., Brabcová I., Bejvančická P. (2020). Risk assessment of falls. Medicína Praxi.

[13] Plati C., Lanara V., Mantas J. (1992). Risk factors responsible for patients’ falls. Scand. J. Caring Sci..

[14] Callis N. (2016). Falls prevention: Identification of predictive fall risk factors. Appl. Nurs. Res..

[15] Najafpour Z., Godarzi Z., Arab M., Yaseri M. (2019). Risk Factors for Falls in Hospital In-Patients: A Prospective Nested Case Control Study. Int. J. Health Policy Manag..

[16] Franck L.S., Gay C.L., Cooper B., Ezrre S., Murphy B., Chan J.S.-L., Buick M., Meer C.R. (2017). The Little Schmidy Pediatric Hospital Fall Risk Assessment Index: A diagnostic accuracy study. Int. J. Nurs. Stud..

[17] Hill-Rodriguez D., Messmer P.R., Williams P.D., Zeller R.A., Williams A.R., Wood M., Henry M. (2009). The Humpty Dumpty Falls Scale: A Case–Control Study. J. Spec. Pediatr. Nurs..

[18] Chromá J. (2016). Risk of falling in pediatric nursing. Cent. Eur. J. Nurs. Midwifery.

[19] Pokorná A., Štrombachová V., Kučerová J., Búřilová P., Dolanová D., Pospíšil M. Metodika Sledování Nežádoucích Událostí ve Zdravotnických Zařízeních Lůžkové Péče 2023. https://shnu.uzis.cz/res/file/metodicke_dokumenty/obecna_metodika_sledovani_nu_2022_final_na_web.pdf.

[20] Lapierre N., Neubauer N., Miguel-Cruz A., Rios Rincon A., Liu L., Rousseau J. (2018). The state of knowledge on technologies and their use for fall detection: A scoping review. Int. J. Med. Inf..

[21] Ren L., Peng Y. (2019). Research of Fall Detection and Fall Prevention Technologies: A Systematic Review. IEEE Access.

[22] Hu X., Qu X. (2016). Pre-impact fall detection. Biomed. Eng. OnLine.

[23] Leone A., Rescio G., Caroppo A., Siciliano P., Andò B., Baldini F., Di Natale C., Marrazza G., Siciliano P. (2018). Wireless Electromyography Technology for Fall Risk Evaluation. Sensors.

[24] Leone A., Rescio G., Caroppo A., Siciliano P. (2015). A Wearable EMG-based System Pre-fall Detector. Procedia Eng..

[25] Rescio G., Leone A., Caroppo A., Casino F., Siciliano P. (2015). A Minimally Invasive Electromyography-based System for Pre-fall Detection. Int. J. Eng. Innov. Technol..

[26] Noury N., Fleury A., Rumeau P., Bourke A.K., Laighin G.O., Rialle V., Lundy J.E. Fall detection—Principles and Methods. Proceedings of the 2007 29th Annual International Conference of the IEEE Engineering in Medicine and Biology Society.

[27] Hong S., Kim H. (2025). Detecting environmental barriers affecting older adult pedestrians via Gramian angular field-based CNN of smartphone sensor data. Front. Public Health.

[28] Leone A., Rescio G., Giampetruzzi L., Siciliano P. (2019). Smart EMG-based Socks for Leg Muscles Contraction Assessment. Proceedings of the 2019 IEEE International Symposium on Measurements & Networking (M&N).

[29] Santoyo-Ramón J.A., Casilari-Pérez E., Cano-García J.M. (2021). A study on the impact of the users’ characteristics on the performance of wearable fall detection systems. Sci. Rep..

[30] Kiprijanovska I., Gjoreski H., Gams M., Kiprijanovska I., Gjoreski H., Gams M. (2020). Detection of Gait Abnormalities for Fall Risk Assessment Using Wrist-Worn Inertial Sensors and Deep Learning. Sensors.

[31] Zhou Y., Zhang D., Ji Y., Bu S., Hu X., Zhao C., Lv Z., Li L. (2025). Wearable sensors and machine learning fusion-based fall risk prediction in covert cerebral small vessel disease. Front. Neurosci..

[32] Butt F.S., La Blunda L., Wagner M.F., Schäfer J., Medina-Bulo I., Gómez-Ullate D. (2021). Fall Detection from Electrocardiogram (ECG) Signals and Classification by Deep Transfer Learning. Information.

[33] Castaldo R., Pecchia L., Kyriacou E., Christofides S., Pattichis C.S. (2016). Preliminary Results from a Proof of Concept Study for Fall Detection via ECG Morphology. Proceedings of the XIV Mediterranean Conference on Medical and Biological Engineering and Computing 2016, Cyprus, 31 March–2 April 2016.

[34] Melillo P., Castaldo R., Sannino G., Orrico A., de Pietro G., Pecchia L. Wearable technology and ECG processing for fall risk assessment, prevention and detection. Proceedings of the 2015 37th Annual International Conference of the IEEE Engineering in Medicine and Biology Society (EMBC) 2015.

[35] Nadeem A., Mehmood A., Rizwan K. (2019). A dataset build using wearable inertial measurement and ECG sensors for activity recognition, fall detection and basic heart anomaly detection system. Data Brief.

[36] Luna-Perejón F., Muñoz-Saavedra L., Civit-Masot J., Civit A., Domínguez-Morales M. (2021). AnkFall—Falls, Falling Risks and Daily-Life Activities Dataset with an Ankle-Placed Accelerometer and Training Using Recurrent Neural Networks. Sensors.

[37] Sudarshan B.G., Hegde R., Kumar P., Satyanarayana B.S. (2013). Design and Development of Fall Detector Using Fall Acceleration. Int. J. Res. Eng. Technol..

[38] Wang Z., Ramamoorthy V., Gal U., Guez A. (2020). Possible Life Saver: A Review on Human Fall Detection Technology. Robotics.

[39] Alves J., Silva J., Grifo E., Resende C., Sousa I., Alves J., Silva J., Grifo E., Resende C., Sousa I. (2019). Wearable Embedded Intelligence for Detection of Falls Independently of on-Body Location. Sensors.

[40] Bourke A.K., O’Brien J.V., Lyons G.M. (2007). Evaluation of a threshold-based tri-axial accelerometer fall detection algorithm. Gait Posture.

[41] Wu G., Xue S. (2008). Portable Preimpact Fall Detector with Inertial Sensors. IEEE Trans. Neural Syst. Rehabil. Eng..

[42] Scheurer S., Koch J., Kucera M., Bryn H., Bärtschi M., Meerstetter T., Nef T., Urwyler P., Scheurer S., Koch J. (2019). Optimization and Technical Validation of the AIDE-MOI Fall Detection Algorithm in a Real-Life Setting with Older Adults. Sensors.

[43] Vallabh P., Malekian R. (2018). Fall detection monitoring systems: A comprehensive review. J. Ambient Intell. Humaniz. Comput..

[44] Er J.K., Ang W.T. Evaluation of Single HMM as a Pre-Impact Fall Detector Based on Different Input Signals. Proceedings of the 2018 IEEE Region Ten Symposium (Tensymp).

[45] Pierleoni P., Belli A., Maurizi L., Palma L., Pernini L., Paniccia M., Valenti S. (2016). A Wearable Fall Detector for Elderly People Based on AHRS and Barometric Sensor. IEEE Sens. J..

[46] Luna-Perejón F., Domínguez-Morales M.J., Civit-Balcells A. (2019). Wearable Fall Detector Using Recurrent Neural Networks. Sensors.

[47] Zhen T., Mao L., Wang J., Gao Q. Wearable preimpact fall detector using SVM. Proceedings of the 2016 10th International Conference on Sensing Technology (ICST).

[48] Vavoulas G., Pediaditis M., Chatzaki C., Spanakis E.G., Tsiknakis M. (2014). The MobiFall Dataset: Fall Detection and Classification with a Smartphone. Int. J. Monit. Surveill. Technol. Res..

[49] Albert M.V., Kording K., Herrmann M., Jayaraman A. (2012). Fall Classification by Machine Learning Using Mobile Phones. PLoS ONE.

[50] Yadav S.K., Tiwari K., Pandey H.M., Akbar S.A. (2022). Skeleton-based human activity recognition using ConvLSTM and guided feature learning. Soft Comput..

[51] Su C., Wei J., Lin D., Kong L., Guan Y.L. (2024). A novel model for fall detection and action recognition combined lightweight 3D-CNN and convolutional LSTM networks. Pattern Anal. Appl..

[52] Doulamis N. (2016). Vision Based Fall Detector Exploiting Deep Learning. Proceedings of the 9th ACM International Conference on PErvasive Technologies Related to Assistive Environments, Corfu Island, Greece, 29 June–1 July 2016.

[53] Zhang Z., Conly C., Athitsos V. (2015). A survey on vision-based fall detection. Proceedings of the 8th ACM International Conference on PErvasive Technologies Related to Assistive Environments, Corfu Island, Greece, 30 June–3 July 2016.

[54] Lee D.-W., Jun K., Naheem K., Kim M.S. (2021). Deep Neural Network–Based Double-Check Method for Fall Detection Using IMU-L Sensor and RGB Camera Data. IEEE Access.

[55] Dutt M., Gupta A., Goodwin M., Omlin C.W., Dutt M., Gupta A., Goodwin M., Omlin C.W. (2024). An Interpretable Modular Deep Learning Framework for Video-Based Fall Detection. Appl. Sci..

[56] Hellmers S., Krey E., Gashi A., Koschate J., Schmidt L., Stuckenschneider T., Hein A., Zieschang T. (2023). Comparison of machine learning approaches for near-fall-detection with motion sensors. Front. Digit. Health.

[57] Joo J.-E., Hu Y., Kim S., Kim H., Park S., Kim J.-H., Kim Y., Park S.-M. (2022). An Indoor-Monitoring LiDAR Sensor for Patients with Alzheimer Disease Residing in Long-Term Care Facilities. Sensors.

[58] Frøvik N., Malekzai B.A., Øvsthus K. (2021). Utilising LiDAR for fall detection. Healthc. Technol. Lett..

[59] Miawarni H., Sardjono T.A., Setijadi E., Wijayanti, Arraziqi D., Gumelar A.B., Purnomo M.H. Fall Detection System for Elderly based on 2D LiDAR: A Preliminary Study of Fall Incident and Activities of Daily Living (ADL) Detection. Proceedings of the 2020 International Conference on Computer Engineering, Network, and Intelligent Multimedia (CENIM).

[60] Bouazizi M., Ye C., Ohtsuki T. (2022). 2-D LIDAR-Based Approach for Activity Identification and Fall Detection. IEEE Internet Things J..

[61] Diraco G., Leone A., Siciliano P., Andò B., Baldini F., Di Natale C., Marrazza G., Siciliano P. (2018). A Fall Detector Based on Ultra-Wideband Radar Sensing. Sensors.

[62] Islam M.M., Tayan O., Islam M.R., Islam M.S., Nooruddin S., Nomani Kabir M., Islam M.R. (2020). Deep Learning Based Systems Developed for Fall Detection: A Review. IEEE Access.

[63] Rezaei A., Mascheroni A., Stevens M.C., Argha R., Papandrea M., Puiatti A., Lovell N.H. (2023). Unobtrusive Human Fall Detection System Using mmWave Radar and Data Driven Methods. IEEE Sens. J..

[64] Mercuri M., Soh P.J., Zheng X., Karsmakers P., Vandenbosch G.A.E., Leroux P., Schreurs D. Analysis of a fall detection radar placed on the ceiling and wall. Proceedings of the 2014 Asia-Pacific Microwave Conference.

[65] Su B.Y., Ho K.C., Rantz M.J., Skubic M. (2015). Doppler Radar Fall Activity Detection Using the Wavelet Transform. IEEE Trans. Biomed. Eng..

[66] Jokanovic B., Amin M.G., Ahmad F. Effect of data representations on deep learning in fall detection. Proceedings of the 2016 IEEE Sensor Array and Multichannel Signal Processing Workshop (SAM).

[67] Liu L., Popescu M., Skubic M., Rantz M., Yardibi T., Cuddihy P. Automatic fall detection based on Doppler radar motion signature. Proceedings of the 2011 5th International Conference on Pervasive Computing Technologies for Healthcare (PervasiveHealth) and Workshops.

[68] Li Z., Du J., Zhu B., Greenwald S.E., Xu L., Yao Y., Bao N., Li Z., Du J., Zhu B. (2024). Doppler Radar Sensor-Based Fall Detection Using a Convolutional Bidirectional Long Short-Term Memory Model. Sensors.

[69] Erol B., Amin M.G. Radar Data Cube Analysis for Fall Detection. Proceedings of the 2018 IEEE International Conference on Acoustics, Speech and Signal Processing (ICASSP).

[70] Ding C., Zou Y., Sun L., Hong H., Zhu X., Li C. Fall detection with multi-domain features by a portable FMCW radar. Proceedings of the 2019 IEEE MTT-S International Wireless Symposium (IWS).

[71] Yang T., Cao J., Guo Y. Placement selection of millimeter wave FMCW radar for indoor fall detection. Proceedings of the 2018 IEEE MTT-S International Wireless Symposium (IWS).

[72] Yao Y., Zhang H., Liu C., Geng F., Wang P., Du L., Chen X., Han B., Yang T., Fang Z. (2024). Unsupervised-Learning-Based Unobtrusive Fall Detection Using FMCW Radar. IEEE Internet Things J..

[73] Ma L., Li X., Liu G., Cai Y., Ma L., Li X., Liu G., Cai Y. (2023). Fall Direction Detection in Motion State Based on the FMCW Radar. Sensors.

[74] Liang T., Liu R., Yang L., Lin Y., Shi C.-J.R., Xu H. (2024). Fall Detection System Based on Point Cloud Enhancement Model for 24 GHz FMCW Radar. Sensors.

[75] Baik J.-Y., Shin H.-C. (2024). Fall Detection Using FMCW Radar to Reduce Detection Errors for the Elderly. J. Electromagn. Eng. Sci..

[76] Cho H., Kang S., Sim Y., Lee S., Jung Y., Cho H., Kang S., Sim Y., Lee S., Jung Y. (2025). Fall Detection Based on Continuous Wave Radar Sensor Using Binarized Neural Networks. Appl. Sci..

[77] Tewari R.C., Sharma S., Routray A., Maiti J. (2023). Effective fall detection and post-fall breath rate tracking using a low-cost CW Doppler radar sensor. Comput. Biol. Med..

[78] Jokanovic B., Amin M., Ahmad F. Radar fall motion detection using deep learning. Proceedings of the 2016 IEEE Radar Conference (RadarConf).

[79] Rodriguez J., Mercuri M., Karsmakers P., Soh P.J., Leroux P., Schreurs D., Pollin S., Van der Perre L., Stas A. (2013). Automatic fall detector based on sliding window principle. Proceedings of the 34th WIC Symposium on Information Theory in the Benelux and the Third joint WIC/IEEE SP Symposium on Information Theory and Signal Processing in the Benelux, Leuven, Belgium, 30–31 May 2013.

[80] Sadreazami H., Bolic M., Rajan S. (2019). CapsFall: Fall Detection Using Ultra-Wideband Radar and Capsule Network. IEEE Access.

[81] Imbeault-Nepton T., Maître J., Bouchard K., Gaboury S. (2023). Fall Detection from UWB Radars: A Comparative Analysis of Deep Learning and Classical Machine Learning Techniques. Proceedings of the 2023 ACM Conference on Information Technology for Social Good, Lisbon, Portugal, 6–8 September 2023.

[82] Wang X., Ellul J., Azzopardi G. (2020). Elderly Fall Detection Systems: A Literature Survey. Front. Robot. AI.

[83] Damodaran N., Haruni E., Kokhkharova M., Schäfer J. (2020). Device free human activity and fall recognition using WiFi channel state information (CSI). CCF Trans. Pervasive Comput. Interact..

[84] Palipana S., Rojas D., Agrawal P., Pesch D. (2018). FallDeFi: Ubiquitous Fall Detection using Commodity Wi-Fi Devices. Proc. ACM Interact. Mob. Wearable Ubiquitous Technol..

[85] Mattela G., Tripathi M., Pal C. (2022). A Novel Approach in WiFi CSI-Based Fall Detection. SN Comput. Sci..

[86] Wang Y., Wu K., Ni L.M. (2017). WiFall: Device-Free Fall Detection by Wireless Networks. IEEE Trans. Mob. Comput..

[87] Shan Z., Li R., Schwertfeger S., Shan Z., Li R., Schwertfeger S. (2019). RGBD-Inertial Trajectory Estimation and Mapping for Ground Robots. Sensors.

[88] Tölgyessy M., Dekan M., Chovanec Ľ., Tölgyessy M., Dekan M., Chovanec Ľ. (2021). Skeleton Tracking Accuracy and Precision Evaluation of Kinect V1, Kinect V2, and the Azure Kinect. Appl. Sci..

[89] Kepski M., Kwolek B., Miesenberger K., Karshmer A., Penaz P., Zagler W. (2012). Fall Detection on Embedded Platform Using Kinect and Wireless Accelerometer. Computers Helping People with Special Needs.

[90] Zobi M., Bolzani L., Tabii Y., Thami R.O.H., Zobi M., Bolzani L., Tabii Y., Thami R.O.H. (2025). Robust 3D Skeletal Joint Fall Detection in Occluded and Rotated Views Using Data Augmentation and Inference–Time Aggregation. Sensors.

[91] Kwolek B., Kepski M. (2014). Human fall detection on embedded platform using depth maps and wireless accelerometer. Comput. Methods Programs Biomed..

[92] Rezaei A.M., Stevens M.C., Argha A., Mascheroni A., Puiatti A., Lovell N.H. An Unobtrusive Fall Detection System Using Low Resolution Thermal Sensors and Convolutional Neural Networks. Proceedings of the 2021 43rd Annual International Conference of the IEEE Engineering in Medicine & Biology Society (EMBC).

[93] Fan X., Zhang H., Leung C., Shen Z. Robust unobtrusive fall detection using infrared array sensors. Proceedings of the 2017 IEEE International Conference on Multisensor Fusion and Integration for Intelligent Systems (MFI).

[94] Nogas J., Khan S.S., Mihailidis A. (2020). DeepFall: Non-Invasive Fall Detection with Deep Spatio-Temporal Convolutional Autoencoders. J. Healthc. Inform. Res..

[95] Alex J.S.R., Abai Kumar M., Swathy D.V., Zhou N., Hemamalini S. (2021). Deep Learning Approaches for Fall Detection Using Acoustic Information. Advances in Smart Grid Technology.

[96] Lian J., Yuan X., Li M., Tzeng N.-F. (2021). Fall Detection via Inaudible Acoustic Sensing. Proc. ACM Interact. Mob. Wearable Ubiquitous Technol..

[97] Alwan M., Rajendran P.J., Kell S., Mack D., Dalal S., Wolfe M., Felder R. A Smart and Passive Floor-Vibration Based Fall Detector for Elderly. Proceedings of the 2006 2nd International Conference on Information & Communication Technologies.

[98] Clemente J., Li F., Valero M., Song W. (2020). Smart Seismic Sensing for Indoor Fall Detection, Location, and Notification. IEEE J. Biomed. Health Inform..

[99] Clemente J., Song W., Valero M., Li F., Liy X. Indoor Person Identification and Fall Detection through Non-intrusive Floor Seismic Sensing. Proceedings of the 2019 IEEE International Conference on Smart Computing (SMARTCOMP).

[100] Okumura N., Yamanoi Y., Kato R., Yamamura O. Fall detection and walking estimation using floor vibration for solitary elderly people. Proceedings of the 2019 IEEE International Conference on Systems, Man and Cybernetics (SMC).

[101] Hassan C.A.U., Karim F.K., Abbas A., Iqbal J., Elmannai H., Hussain S., Ullah S.S., Khan M.S. (2023). A Cost-Effective Fall-Detection Framework for the Elderly Using Sensor-Based Technologies. Sustainability.

[102] Lin Y., Zhao Q., Lin Y., Zhao Q. (2024). Human Occupancy Monitoring and Positioning with Speed-Responsive Adaptive Sliding Window Using an Infrared Thermal Array Sensor. Sensors.

[103] He C., Liu S., Zhong G., Wu H., Cheng L., Yan G., Wen Y. (2023). A Noncontact Fall Detection Method for Bedside Application With a MEMS Infrared Sensor and a Radar Sensor. IEEE Internet Things J..

[104] Yun J., Lee S.-S., Yun J., Lee S.-S. (2014). Human Movement Detection and Identification Using Pyroelectric Infrared Sensors. Sensors.

[105] He C., Liu S., Zhong G., Wu H., Cheng L., Lin J., Huang Q. (2023). A Non-Contact Fall Detection Method for Bathroom Application Based on MEMS Infrared Sensors. Micromachines.

[106] Chen W., Jiang Z., Guo H., Ni X. (2020). Fall Detection Based on Key Points of Human-Skeleton Using OpenPose. Symmetry.

[107] Liu J., Lockhart T.E. (2009). Trunk Angular Kinematics during Slip-Induced Falls and Activities of Daily Living—Towards Developing a Fall Detector. Proc. Hum. Factors Ergon. Soc. Annu. Meet..

[108] Yu X., Jang J., Xiong S. (2021). A Large-Scale Open Motion Dataset (KFall) and Benchmark Algorithms for Detecting Pre-impact Fall of the Elderly Using Wearable Inertial Sensors. Front. Aging Neurosci..

[109] Hauth J., Jabri S., Kamran F., Feleke E.W., Nigusie K., Ojeda L.V., Handelzalts S., Nyquist L., Alexander N.B., Huan X. (2021). Automated Loss-of-Balance Event Identification in Older Adults at Risk of Falls during Real-World Walking Using Wearable Inertial Measurement Units. Sensors.

[110] Cartocci N., Gkikakis A.E., Kurvina N., Takele N., Pera F., Settino M.T., Caldwell D.G., Ortiz J., Jin S., Kim J.H., Kong Y.-K., Park J., Yun M.H. (2025). Recognition of Physiological Patterns During Activities of Daily Living Using Wearable Biosignal Sensors. Proceedings of the 22nd Congress of the International Ergonomics Association, Volume 3, Jeju, Republic of Korea, 25–29 August 2024.

[111] Palmerini L., Klenk J., Becker C., Chiari L., Palmerini L., Klenk J., Becker C., Chiari L. (2020). Accelerometer-Based Fall Detection Using Machine Learning: Training and Testing on Real-World Falls. Sensors.

[112] Chaudhuri S., Oudejans D., Thompson H.J., Demiris G. (2015). Real World Accuracy and Use of a Wearable Fall Detection Device by Older Adults. J. Am. Geriatr. Soc..

[113] Uddin M.Z., Khaksar W., Torresen J., Uddin M.Z., Khaksar W., Torresen J. (2018). Ambient Sensors for Elderly Care and Independent Living: A Survey. Sensors.

[114] Hsu F.-S., Chang T.-C., Su Z.-J., Huang S.-J., Chen C.-C. (2021). Smart Fall Detection Framework Using Hybridized Video and Ultrasonic Sensors. Micromachines.

[115] Kibet D., So M.S., Kang H., Han Y., Shin J.-H. (2024). Sudden Fall Detection of Human Body Using Transformer Model. Sensors.

[116] Núñez-Marcos A., Arganda-Carreras I. (2024). Transformer-based fall detection in videos. Eng. Appl. Artif. Intell..

[117] Zafar R.O., Zafar F. (2025). Real-time activity and fall detection using transformer-based deep learning models for elderly care applications. BMJ Health Care Inform..

[118] Shin J., Hassan N., Miah1 A.S.M., Nishimura S. (2024). A Comprehensive Methodological Survey of Human Activity Recognition Across Divers Data Modalities 2024. arXiv.

[119] Bian S., Liu M., Zhou B., Lukowicz P., Bian S., Liu M., Zhou B., Lukowicz P. (2022). The State-of-the-Art Sensing Techniques in Human Activity Recognition: A Survey. Sensors.

[120] Gomaa W., Khamis M.A. (2023). A perspective on human activity recognition from inertial motion data. Neural Comput. Appl..

[121] Gaya-Morey F.X., Manresa-Yee C., Buades-Rubio J.M. (2024). Deep Learning for Computer Vision based Activity Recognition and Fall Detection of the Elderly: A Systematic Review. Appl. Intell..

[122] Ciortuz G., Hozhabr Pour H., Irshad M.T., Nisar M.A., Huang X., Fudickar S. (2025). Machine learning models for wearable-based human activity recognition: A comparative study. Neurocomputing.

[123] Attal F., Mohammed S., Dedabrishvili M., Chamroukhi F., Oukhellou L., Amirat Y., Attal F., Mohammed S., Dedabrishvili M., Chamroukhi F. (2015). Physical Human Activity Recognition Using Wearable Sensors. Sensors.

[124] Siwadamrongpong W., Chinrungrueng J., Hasegawa S., Nantajeewarawat E. Fall Detection and Prediction Based on IMU and EMG Sensors for Elders. Proceedings of the 2022 19th International Joint Conference on Computer Science and Software Engineering (JCSSE).

[125] Yan J., Wang X., Shi J., Hu S. (2023). Skeleton-Based Fall Detection with Multiple Inertial Sensors Using Spatial-Temporal Graph Convolutional Networks. Sensors.

[126] Tian Z., Zhang L., Wang G., Wang X. (2022). An RGB camera-based fall detection algorithm in complex home environments. Interdiscip. Nurs. Res..

[127] Liu H., Liu T., Chen Y., Zhang Z., Li Y.F. (2024). EHPE: Skeleton Cues-Based Gaussian Coordinate Encoding for Efficient Human Pose Estimation. IEEE Trans. Multimed..

[128] Liu T., Liu H., Yang B., Zhang Z. (2024). LDCNet: Limb Direction Cues-Aware Network for Flexible HPE in Industrial Behavioral Biometrics Systems. IEEE Trans. Ind. Inform..

[129] Liu H., Chen Q., Liu Z., Liu T., Zhao L., Zhang Z., Li Y.F. (2025). SkeFormer: Skeletal Cues-aware Bone point Relationship Learning for Efficient FBIC via Transformers. IEEE Trans. Multimed..

[130] Ahn S., Choi M., Lee J., Kim J., Chung S. (2025). Non-Contact Fall Detection System Using 4D Imaging Radar for Elderly Safety Based on a CNN Model. Sensors.

[131] Aziz O., Klenk J., Schwickert L., Chiari L., Becker C., Park E.J., Mori G., Robinovitch S.N. (2017). Validation of accuracy of SVM-based fall detection system using real-world fall and non-fall datasets. PLoS ONE.

[132] Bagalà F., Becker C., Cappello A., Chiari L., Aminian K., Hausdorff J.M., Zijlstra W., Klenk J. (2012). Evaluation of Accelerometer-Based Fall Detection Algorithms on Real-World Falls. PLoS ONE.

[133] Ghayvat H., Pandya S., Patel A. Proposal and Preliminary Fall-related Activities Recognition in Indoor Environment. Proceedings of the 2019 IEEE 19th International Conference on Communication Technology (ICCT).

[134] Harari Y., Shawen N., Mummidisetty C.K., Albert M.V., Kording K.P., Jayaraman A. (2021). A smartphone-based online system for fall detection with alert notifications and contextual information of real-life falls. J. NeuroEngineering Rehabil..

[135] Stampfler T., Elgendi M., Fletcher R.R., Menon C. (2022). Fall detection using accelerometer-based smartphones: Where do we go from here?. Front. Public Health.

[136] Lin H.-C., Chen M.-J., Lee C.-H., Kung L.-C., Huang J.-T. (2023). Fall Recognition Based on an IMU Wearable Device and Fall Verification through a Smart Speaker and the IoT. Sensors.

[137] Hu S., Cao S., Toosizadeh N., Barton J., Hector M.G., Fain M.J. (2024). Radar-Based Fall Detection: A Survey. IEEE Robot. Autom. Mag..

[138] Nadee C., Chamnongthai K. (2022). An Ultrasonic-Based Sensor System for Elderly Fall Monitoring in a Smart Room. J. Healthc. Eng..

[139] Cardenas J.D., Gutierrez C.A., Aguilar-Ponce R. (2021). Influence of the Antenna Orientation on WiFi-Based Fall Detection Systems. Sensors.

[140] Ji S., Xie Y., Li M. (2023). SiFall: Practical Online Fall Detection with RF Sensing. Proceedings of the 20th ACM Conference on Embedded Networked Sensor Systems, Boston, MA, USA, 6–9 November 2022.

[141] Nizam Y., Mohd M.N.H., Jamil M.M.A. (2017). Human Fall Detection from Depth Images using Position and Velocity of Subject. Procedia Comput. Sci..

[142] Yang J., He Y., Zhu J., Lv Z., Jin W. (2024). Fall Detection Method for Infrared Videos Based on Spatial-Temporal Graph Convolutional Network. Sensors.

[143] Kaur P., Wang Q., Shi W. (2022). Fall detection from audios with Audio Transformers. Smart Health.

[144] Shao Y., Wang X., Song W., Ilyas S., Guo H., Chang W.-S. (2020). Feasibility of Using Floor Vibration to Detect Human Falls. Int. J. Environ. Res. Public. Health.

[145] Salimi M., Machado J.J.M., Tavares J.M.R.S. (2022). Using Deep Neural Networks for Human Fall Detection Based on Pose Estimation. Sensors.

[146] Lin C.-B., Dong Z., Kuan W.-K., Huang Y.-F. (2020). A Framework for Fall Detection Based on OpenPose Skeleton and LSTM/GRU Models. Appl. Sci..

[147] Yu X., Koo B., Jang J., Kim Y., Xiong S. (2022). A comprehensive comparison of accuracy and practicality of different types of algorithms for pre-impact fall detection using both young and old adults. Measurement.

[148] Skubic M., Harris B.H., Stone E., Ho K.C., Su B.-Y., Rantz M. Testing non-wearable fall detection methods in the homes of older adults. Proceedings of the 2016 38th Annual International Conference of the IEEE Engineering in Medicine and Biology Society (EMBC).

[149] Casilari E., Lora-Rivera R., García-Lagos F. (2020). A Study on the Application of Convolutional Neural Networks to Fall Detection Evaluated with Multiple Public Datasets. Sensors.

[150] Chelli A., Pätzold M. (2019). A Machine Learning Approach for Fall Detection Based on the Instantaneous Doppler Frequency. IEEE Access.

[151] Wang X., Ma P., Lian J., Liu J., Ma Y. (2025). An echo state network based on enhanced intersecting cortical model for discrete chaotic system prediction. Front. Phys..

